# Reducing referrals in primary care through specialist-supported telehealth: quasi-experimental evidence from Northeast Brazil

**DOI:** 10.3389/fdgth.2026.1890968

**Published:** 2026-09-09

**Authors:** Frederica Valle de Queiroz Padilha, Francisco Antônio Sousa de Araújo, Jorge Norio Rezende Ikawa, Maria Eulália Vinadé Chagas, Natalia Luiza Kops, Fernanda Saks Hahne, Vivian Oliveira Balan

**Affiliations:** 1 Hospital Alemão Oswaldo Cruz, São Paulo, Brazil; 2 BP- A Beneficência Portuguesa de São Paulo, São Paulo, Brazil; 3 Hospital do Coração, São Paulo, Brazil; 4 Hospital Moinhos de Vento, Porto Alegre, Brazil

**Keywords:** impact evaluation, primary care referral rates, telehealth, tele-interconsultations, workplace learning

## Abstract

**Background:**

Synchronous tele-interconsultations — real-time interactions linking primary care physicians and hospital-based specialists — have been proposed as a way to support clinical decision-making and, potentially, to build clinical competencies over time through repeated exposure to specialist reasoning. However, causal evidence on whether such programs reduce reliance on specialist referrals remains limited, particularly in low- and middle-income country settings characterized by large territories and uneven specialist distribution.

**Methods:**

We conducted a quasi-experimental evaluation of the TeleNordeste project, a large-scale telehealth initiative implemented in Northeast Brazilian states under a public-private partnership. Using staggered difference-in-differences methods, our primary aim was to estimate the treatment effect on primary care referral rates, using a panel of primary care units observed from 2018 through 2025. After applying a municipality-level restriction to reduce spillover contamination, the analytical sample comprised 6,825 primary care units: 3,918 never-treated comparison units, 1,002 low-dose units (1–4 cumulative telehealth interactions), and 1,905 high-dose units (≥5 interactions). Secondary analyses explored whether estimated effects were more consistent with immediate care-flow reorganization or workplace learning.

**Results:**

Any exposure to the program was associated with a statistically significant reduction in referral rates (−0.356 percentage points, no-covariate specification; *p* < 0.001). Similar estimates were observed across low- and high-exposure groups, with no evidence that effects increased with cumulative exposure.

**Conclusions:**

Participation in the TeleNordeste program was associated with meaningful reductions in referrals to specialized care. Although immediate case resolution during tele-interconsultations provides a plausible explanation for this association, workplace learning cannot be confirmed or excluded with the available data. In the absence of a clear dose-response gradient, these mechanisms cannot be disentangled, and the results indicate that program participation itself can strengthen primary care case resolution and reduce reliance on specialist referrals. Interpretation of mechanisms is limited by potential under-registration of referrals and by the use of primary care units, rather than individual physicians, as the unit of analysis. Future evaluations would benefit from collecting physician-level identifiers and strengthening audits of referral-registration completeness, thereby enabling a more detailed assessment of how tele-interconsultations affect clinical practice and referral decisions over time.

## 1 Introduction

Digital health and telemedicine have become central components of health system reform agendas worldwide, driven by the recognition that geography, workforce shortages, and care fragmentation continue to limit the reach and quality of health services — particularly in low- and middle-income countries (1, 2). As these technologies have matured, the research focus has increasingly shifted from documenting access gains to understanding how different modalities shape care quality, clinical workflows, and the professional development of health workers (3, 4). This reframing is especially relevant for primary healthcare (PHC), where the integration of specialist input through digital channels has the potential to go beyond individual patient encounters — influencing how primary care physicians reason, decide, and practice over time (5).

Telehealth encompasses diverse modalities, including synchronous and asynchronous communication between professionals and patients (6). While some primarily facilitate information exchange, others integrate specialist input directly into the clinical encounter, offering distinct types of clinical support and learning opportunities. Professional-to-professional modalities, such as joint specialist encounters, have been associated with improved diagnostic confidence and problem-solving capacity, especially where specialist availability is limited (7, 8).

Synchronous tele-interconsultations—bringing together primary care physicians, specialists, and patients in real time—offer a particularly conducive environment for collaborative learning. Through case-based interactions, primary care physicians gain exposure to specialist reasoning, complementing formal training (4, 9). Systematic reviews suggest such interactions can lead to sustained behavioral changes and greater autonomy in managing cases (10). For instance, Liddy et al. (11) demonstrated that electronic consultations in Canada are associated with lower referral rates, indicating that specialist input can fundamentally alter primary care decision-making.

In Brazil, evaluations of teleconsultation initiatives have reported improvements in primary care practice, including enhanced clinical support and reductions in referrals to specialized care (12, 13). However, most of these studies rely on descriptive or cross-sectional designs, providing limited evidence on causal effects and on how changes in clinical practice unfold over time.

This study addresses this gap by examining the TeleNordeste project, a large-scale telehealth initiative implemented in Brazil's Northeast region under the Program for Institutional Development of the Unified Health System (PROADI-SUS), a national public–private partnership through which projects are developed by private hospitals in collaboration with the Ministry of Health and the National Council of Health Secretaries (14).

The primary aim is to estimate the causal effect of TeleNordeste participation on primary care referral rates to specialized services, using a quasi-experimental design that exploits the staggered rollout of the program across primary care units. As a secondary aim, we use variation in exposure intensity and comparison-group definitions across several model specifications to explore two candidate mechanisms through which such an effect could operate: an immediate reorganization of care flows, whereby cases that would otherwise be referred are resolved through the tele-interconsultation itself, and workplace learning, whereby repeated exposure to specialist reasoning gradually strengthens primary care physicians' diagnostic confidence and case-management capacity. While an immediate reorganization of care flows offers a plausible and sufficient explanation for the effect we estimate, our data do not allow us to confirm whether workplace learning also contributes. The results provide empirical evidence on the potential outcomes of synchronous telemedicine services beyond immediate treatment effects, with insights applicable to similar contexts in countries with large territorial areas and uneven distributions of medical specialists. To our knowledge, this is among the first quasi-experimental evaluations of synchronous specialist-supported telehealth on primary care referral behavior in a LMIC setting.

## 2 Methods

### 2.1 Data sources

The analysis relies on multiple data sources integrated at the primary care unit level. The main source is administrative data from the Primary Care Health Information System (Sistema de Informação em Saúde para a Atenção Básica - SISAB), which consolidates data reported through the e-SUS APS strategy and provides information on individual primary care encounters, including professional category and clinical conduct.

To characterize exposure to the intervention, we used administrative monitoring reports from the TeleNordeste project, provided by the participating hospitals responsible for delivering telehealth services. These reports contain detailed records on the number, timing, and modality of tele-interconsultations conducted by each primary care unit, allowing precise measurement of treatment intensity over time.

Two time-varying covariates were derived from the Cadastro Nacional de Estabelecimentos de Saúde (CNES) workforce registry: (i) the number of physicians registered at each primary care unit in a given quarter, calculated as the monthly average within the quarter; and (ii) physician turnover, measured as the monthly average of the share of physicians who exited the unit within the quarter. Both covariates are included in robustness specifications to improve precision and account for observable changes in workforce composition over time that may confound comparisons between treated and comparison units.

### 2.2 The TeleNordeste intervention

Brazil's Northeast region, comprising approximately 55 million inhabitants across nine states, remains marked by persistent regional inequalities in socioeconomic conditions and health system capacity. Despite substantial expansion of primary healthcare service, the region continues to face challenges related to physician distribution, specialist retention, and geographic access to care (15, 16).

In this context, telehealth initiatives have emerged as a strategic policy response to mitigate geographic barriers and strengthen care delivery in underserved territories (16, 17). TeleNordeste project aims to expand access to specialized care while simultaneously strengthening clinical competencies within primary healthcare units by using telehealth as a structured mechanism for clinical support and knowledge exchange.

This model relies on tele-interconsultations conducted jointly by primary care physicians and hospital-based specialists, with or without the patient participation, depending on the clinical context. Both modalities are designed to support timely clinical decision-making while fostering continuous professional development through repeated exposure to specialist expertise. Telehealth services were delivered by five non-profit private hospitals of excellence participating in the PROADI-SUS program — four based in São Paulo and one in Rio Grande do Sul — each responsible for providing teleconsultation services to primary care units in one, two or three Northeast states within their designated area of coverage. No formal minimum number of sessions was mandated at the unit level; engagement was demand-driven, with primary care physicians initiating consultations based on clinical need. No financial incentives or structured adherence protocols specifically targeting physician participation were in place beyond the institutional support provided through the PROADI-SUS framework.

**Figure 1:**
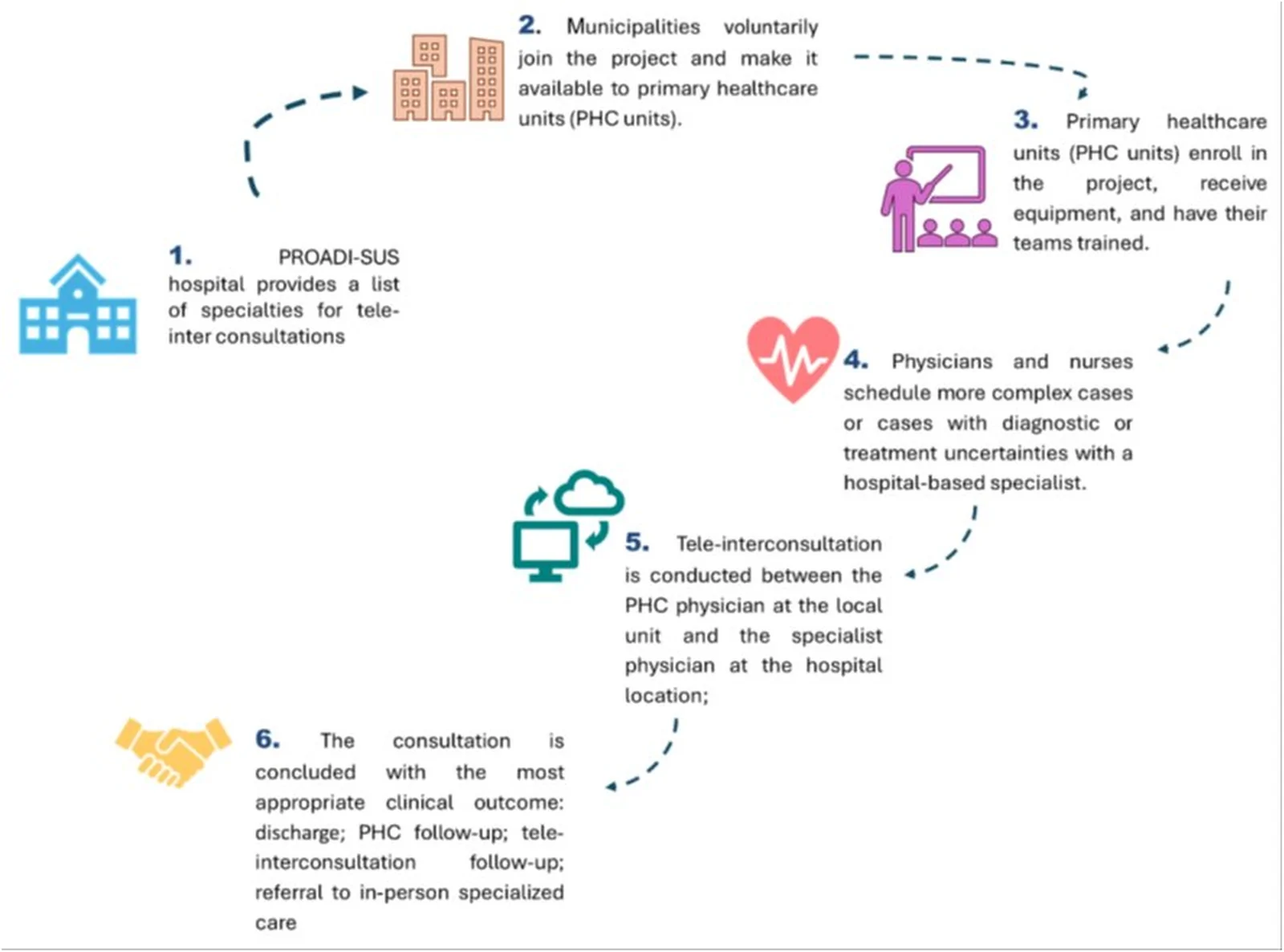
Operational workflow of the TeleNordeste telehealth intervention within the Unified Health System (SUS). Steps illustrate the sequential process from PROADI-SUS hospital enrollment to clinical encounter resolution, including specialist scheduling, tele-interconsultation, and possible outcomes (discharge, primary care follow-up, tele-interconsultation follow-up, or referral to in-person specialized care).

The project's operational workflow is illustrated in Figure 1.

**Figure 2:**
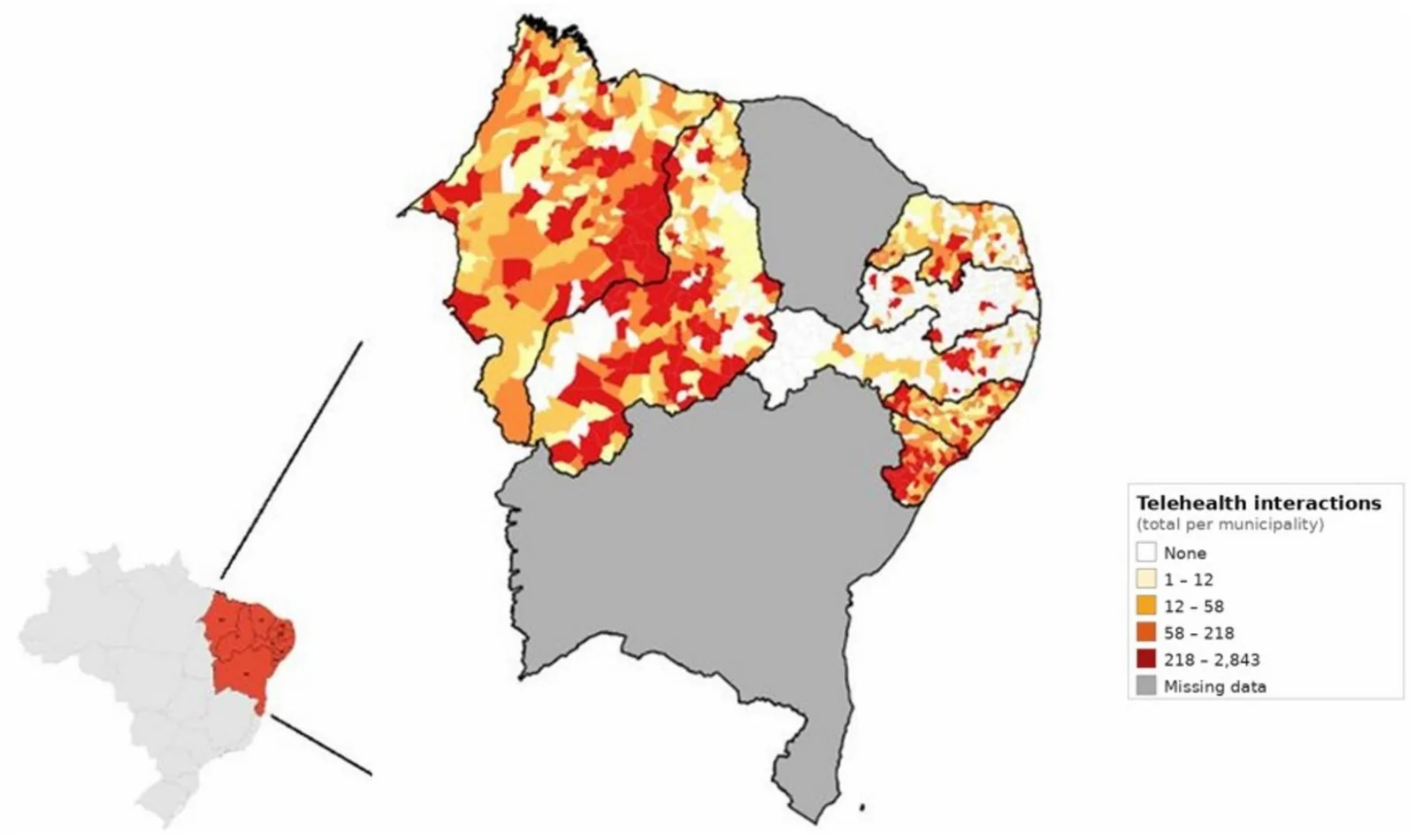
Geographic distribution of telehealth interactions under the TeleNordeste project, by municipality (Northeast Brazil, excluding Ceará and Bahia). Colors represent total cumulative telehealth interactions per municipality across the study period. Gray areas indicate states excluded from the analysis due to unavailability of standardized monitoring data.

While telehealth activity was present in all participating states, intensity varied substantially, with some areas exhibiting clusters of high engagement and others showing sparse or no recorded use. Figure 2 maps the spatial distribution of telehealth exposure at the municipal level, highlighting pronounced heterogeneity across states within the Northeast region. This pattern suggests that local institutional capacity, managerial support, and professional engagement played an important role in shaping the uptake of telehealth services, beyond the mere availability of the intervention. Descriptive evidence from participating states suggests that the project reached a substantial share of the primary care population in the region, including previously underserved groups (18, 19).

In two states — Ceará and Bahia — telehealth activities were implemented under a hospital which did not participate in this study and consequently did not provide standardized administrative and monitoring data; these two states were therefore excluded from the analysis.

### 2.3 Study design

We adopted a quasi-experimental design to estimate the effect of exposure to the TeleNordeste program on referral rates to specialized care, using a staggered difference-in-differences (DiD) approach (20) that exploits variation in the timing and intensity of program exposure across primary care units. Under this design, treatment effects are identified from within-unit changes in referral rates following program exposure, relative to contemporaneous changes among units never exposed during the study period; never-treated units contribute to identification throughout the study period, while treated units contribute observations both before and after program exposure according to their treatment timing.

We defined treatment timing and exposure intensity separately. Exposure intensity reflects a unit's realized dose, based on the total number of tele-interconsultations it accumulated over the full observation window — following precedent for treating cumulative utilization counts as a dose measure (21) — classified into a low-dose group (1–4 interactions) and a high-dose group (≥5 interactions). Categorical grouping was preferred over a continuous specification to ease interpretation and limit sensitivity to the extreme right-skew in cumulative interactions. Because exposure intensity is realized over the full window rather than assigned at baseline, it conditions on post-treatment information and does not affect the temporal identification strategy of the DiD design; it serves only to stratify the analysis by realized exposure intensity, and dose-specific estimates should accordingly be interpreted as comparisons across realized intensity rather than as causal effects of incremental tele-interconsultation volume.

**Figure 3:**
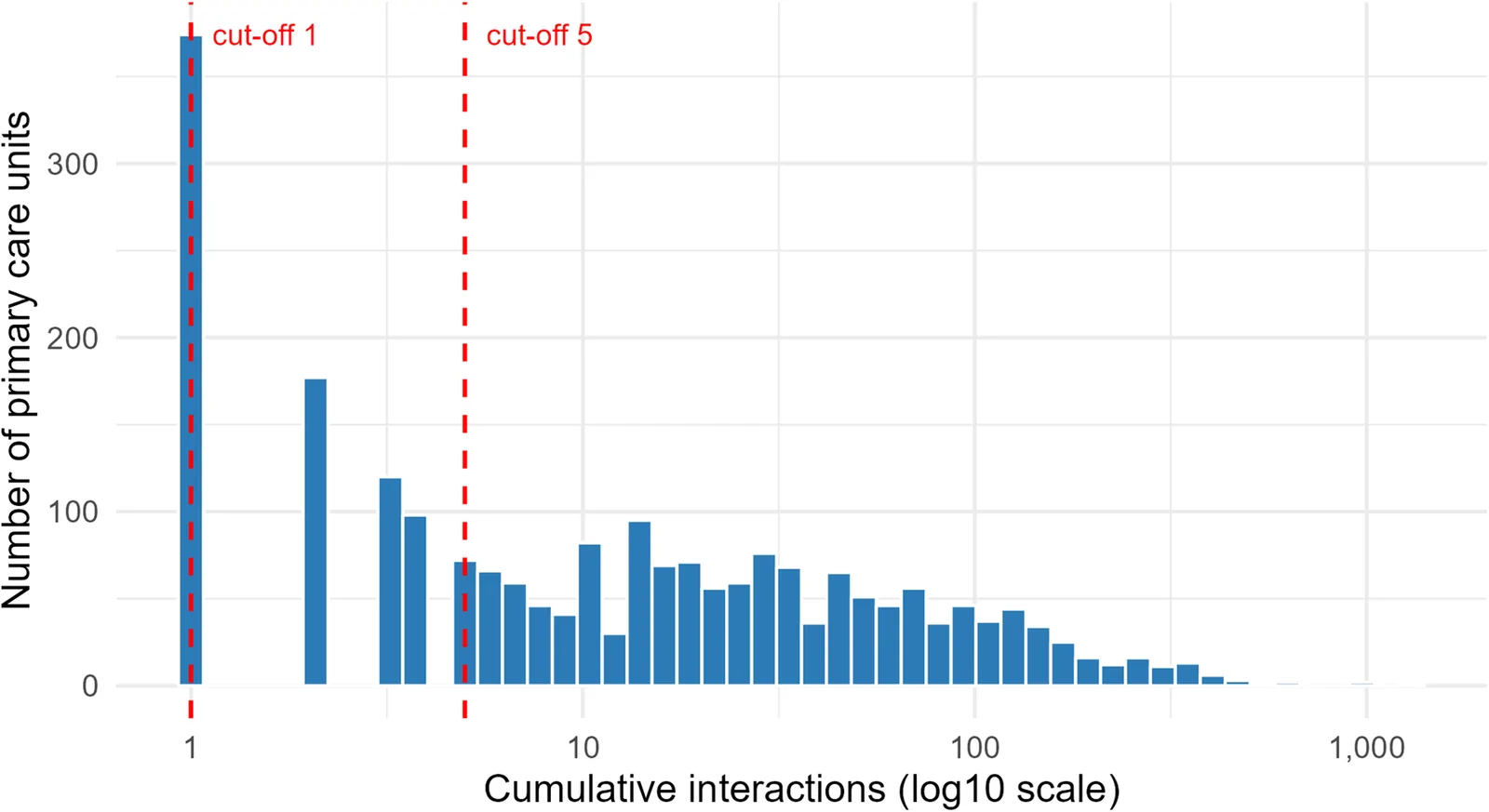
Distribution of cumulative telehealth interactions among exposed primary care units, TeleNordeste project (Northeast Brazil, excluding Ceará and Bahia). The *x*-axis is displayed on a log10 scale. Red dashed lines indicate the cut-off values used in the main models (cut-off 1 and cut-off 5), corresponding to the thresholds separating the low-dose (1–4 interactions) and high-dose (≥5 interactions) groups.

The threshold of five cumulative interactions separating the two dose groups was selected based on the empirical distribution of exposure across primary care units, which exhibits a visible discontinuity around this value consistent with a distinction between incidental and sustained engagement (Figure 3). We assessed the sensitivity of the results to this choice using alternative thresholds of three and seven interactions, estimating both dose groups at each threshold (Supplementary Appendix Table A1).

The comparison group comprises units located in municipalities where no facility ever received tele-interconsultations, limiting the risk of local spillover; the construction of this restriction is detailed below.

Treatment timing, by contrast, concerns when a unit's DiD treatment date is set, independent of its exposure-intensity group. In our main specification, this date is set at the quarter of a unit's first recorded tele-interconsultation for both dose groups. Because a high-dose unit is not high-dose at that first interaction — it only reaches that classification once cumulative interactions later cross the threshold — we additionally estimate a specification in which the treatment date for the high-dose group is instead re-dated at the quarter cumulative interactions first reach five. The two rules diverge for a substantial share of units: 55% of high-dose units required more than one quarter beyond their first interaction to cross the threshold (median additional lag of one quarter; 90th percentile, four quarters). Both rules are reported throughout the Results.

#### 2.3.1 Sample construction and data quality

The analytical sample was constructed from raw SISAB and TeleNordeste monitoring records for the seven included states, comprising 10,401 primary care units before any exclusion, observed from 2018 through Q2 2025. SISAB provides nationwide coverage and standardized reporting fields, but its reliability is subject to known limitations in the Brazilian context, including heterogeneity in data entry practices across municipalities and underreporting of clinical conducts, particularly in regions with lower administrative capacity (22, 23).

Two exclusion steps were applied. First, to guard against structural zeros unrelated to true clinical behavior, we excluded 431 units whose referral count is zero in every observed quarter; zero-referral values in individual quarters of otherwise-reporting units are retained as valid observations, since SISAB does not allow us to distinguish a quarter in which no case required referral from one in which referrals went unrecorded, and excluding these quarters would risk removing genuine information about program effects along with data-quality noise, in either direction. Second, a municipality-level restriction excluded units with no recorded tele-interconsultation exposure that are located in municipalities where at least one other facility did receive them, since these units cannot serve as clean comparison units; this restriction defines the comparison group introduced above.

The resulting analytical sample comprises 6,825 primary care units: 3,918 in the never-treated comparison group, 1,002 in the low-dose group, and 1,905 in the high-dose group.

As a robustness check on the zero-referral rule above, we constructed a data-quality proxy from each unit's zero-referral rate in the pre-rollout period — before Q3 2022, when the program did not yet exist and could not have caused genuine zero-referral quarters — grouping units into six tiers from 0% (perfect reporting) to 76%–100%, and re-estimated our models after excluding units in the worst tiers, applying the same rule to treated and comparison units alike to avoid introducing new selection on treatment status.

The primary outcome is the referral rate to specialized care, defined as the proportion of medical consultations in which the recorded clinical conduct corresponds to referral to specialized services, calculated for each primary care unit and quarter as the ratio of referrals to total medical consultations. No secondary outcomes were pre-specified.

### 2.4 Empirical strategy

Our empirical strategy was designed to estimate the Average Treatment Effect on the Treated (ATT) for specific cohorts of primary care units. Let cohort *g* comprise units first treated in period *g*. The parameter of interest for cohort *g* at time *t* is:

$$ATT(g,\;t)\;=\;E[Y_{t}(g)\;-\;Y_{t}(\infty )\;|\;G\;=\;g]$$

where *Y(g)_t_* denotes the potential outcome of units in cohort *g* at time *t* under treatment, and *Y(0)_t_* represents the counterfactual outcome for units that are never treated. ATTs are aggregated across cohorts and periods using the estimator proposed by Callaway and Sant'Anna (20).

Identification relies on three assumptions: (i) parallel trends — that, in the absence of the intervention, treated units would have followed a trajectory similar to that of never-treated units, either unconditionally in our primary no-covariate specification or conditional on observed covariates in the secondary specifications described below; (ii) no anticipation — that units do not adjust behavior prior to treatment onset; and (iii) treatment irreversibility — that units remain treated once exposure begins.

We adopted four complementary strategies to assess the plausibility of these assumptions in the absence of randomization. The comparison group is restricted to primary care units in municipalities where no facility ever recorded telehealth activity, reducing contamination from local spillovers. Event-study estimates provide an empirical assessment of the parallel trends assumption: pre-treatment coefficients statistically indistinguishable from zero are consistent with this assumption. Two time-varying workforce covariates — number of physicians and physician turnover rate, potential determinants of referral behavior independent of telehealth exposure — were included in alternative specifications to improve comparability and assess sensitivity to observable confounders. Finally, for our three primary no-covariate specifications (M1, M2, M2b), we additionally assess robustness to violations of the parallel trends assumption itself using the “honest” confidence-interval approach of Rambachan and Roth (24), under the relative-magnitude restriction they propose, and report the breakdown value for each specification; we return to the interpretation of these values in the Discussion.

We estimate four complementary specifications, each under three covariate configurations (no covariates; conditioning on the number of physicians; conditioning on physician turnover rate). M1 (Binary) compares any exposed unit (≥1 interaction) against never-treated units, providing an overall average effect. M2 (Dose-response) estimates separate ATTs for the low-dose and high-dose groups, each against never-treated units, with treatment timing set at first interaction. M2b re-estimates the high-dose model with treatment timing set at the threshold-crossing quarter instead. M3 (Incremental) compares high-dose units directly against low-dose units, with treatment timing set at threshold crossing, characterizing the additional association of sustained relative to limited engagement — our most direct test of whether effects scale with cumulative dose, consistent with a cumulative-learning mechanism.

Event-study estimates are reported for ten pre-treatment and nine post-treatment quarters (e = −10 to e = 9); this window retains at least 150 contributing units at every event-time within this range, thereby avoiding estimates based on only a small number of units.

Statistical inference is based on bootstrap procedures with 999 replications, clustered at the primary care unit level; all models allow for unbalanced panels. All bootstrap procedures used a fixed random seed, and estimated ATTs for the three primary no-covariate specifications were robust to the choice of random seed across multiple alternative seeds tested. Estimation is conducted using the *did* package (version 2.1.0) in R (version 4.4.1).

This study used exclusively secondary, anonymized administrative data aggregated at the primary care unit level, with no access to individual-level or personally identifiable information. Due to the nature of the data used, ethical approval was not required under Brazilian Resolution CNS 510/2016, which exempts research conducted solely with anonymized secondary data.

## 3 Results

### 3.1 Baseline characteristics

Between program rollout (Q3 2022) and the end of the observation window (Q2 2025), 125,946 telehealth interactions were recorded across the 2,907 primary care units that received at least one tele-interconsultation, drawn from the full analytical sample of 6,825 units. Telehealth activity was concentrated in a limited set of clinical specialties, most notably endocrinology, neurology, and cardiology.

Table 1 presents pre-treatment characteristics of the three study groups. Although never-treated units exhibit somewhat higher baseline referral rates than either dose group (5.00% versus 3.53% and 3.92%), this may be consistent with positive selection into the program, among other possible explanations, if more institutionally engaged facilities were both more likely to adopt telehealth and more resolutive at baseline. The geographic distribution differs substantially between groups, reflecting the uneven rollout of TeleNordeste and reinforcing the importance of the municipality-level control group restriction.

**Table 1:** Baseline characteristics of primary care units by study group.

Characteristic	Never-treated (*n* = 3,918)	Low-dose (1–4) (*n* = 999)	High-dose (≥5) (*n* = 1,896)
Primary outcome			
Mean Referral rate, % (SD)	5.00 (6.48)	3.53 (5.36)	3.92 (5.32)
Medical consultations per quarter, mean (SD)	672 (477)	597 (559)	688 (598)
Referrals per quarter, mean (SD)	38.1 (59.6)	28.2 (67.2)	33.5 (67.5)
Workforce covariates (available in ∼22%–29% of unit-quarters)			
Number of physicians, mean (SD)	1.28 (0.82)	1.92 (1.97)	1.87 (1.87)
Physician turnover (% exit/month), mean (SD)	6.11 (17.32)	7.33 (18.12)	7.48 (17.85)
Covariate coverage, % of unit-quarters	28.7	22.2	22.3
State distribution, %			
Alagoas (AL)	0.5	20.8	16.1
Maranhão (MA)	5.0	48.9	31.1
Paraíba (PB)	28.2	1.9	4.3
Pernambuco (PE)	51.9	2.7	6.5
Piauí (PI)	5.1	14.0	18.6
Rio Grande do Norte (RN)	8.8	5.7	10.8
Sergipe (SE)	0.5	6.1	12.6

Baseline characteristics are calculated over the pre-treatment period for each unit, defined as all quarters prior to first recording any telehealth activity; for never-treated units, all observed quarters are used. Group *n* reflects units with at least one pre-treatment quarter available (units treated from their first observed quarter contribute no pre-treatment data and are therefore excluded from this table only, not from the analytical sample). SD, standard deviation.

Among treated units, the median cumulative exposure was 12 interactions and the 90th percentile was 122. This right-skewed distribution (Figure 3) shows that exposure was highly uneven — a large share of units engaged minimally, while a smaller subset accumulated repeated interactions — reinforcing the importance of modeling treatment as a cumulative, heterogeneous process rather than a binary indicator.

### 3.2 Main treatment effects

Table 2 reports ATT estimates across four model specifications, each under three covariate configurations. M1 (Binary) compares any exposed unit (≥1 interaction) against never-treated units; M2 (Dose-response) reports separate estimates for the low-dose and high-dose groups against never-treated units; M2b re-estimates the high-dose model with onset re-dated at threshold crossing; M3 (Incremental) compares high-dose units directly against low-dose units. Under the no-covariate configuration, all models except M3 yield statistically significant, negative estimates: any exposure (M1) is associated with a −0.356 percentage-point reduction in referral rates (*p* < 0.001); low-dose and high-dose units show estimates of similar magnitude (−0.335 and −0.365 percentage points, respectively; both *p* < 0.05); and the onset-re-dated high-dose specification (M2b) yields −0.266 percentage points (*p* = 0.017). M3 is small, positive, and not statistically significant (+0.142 percentage points, *p* = 0.236).

**Table 2:** Effect of TeleNordeste on referral rates to specialized care.

Model	Exposure group	Covariate	ATT (p.p.)	95% CI	Relative change	Pre-trends p
M1	Any exposure (≥1)	None	−0.356*	[−0.555, −0.156]	−9.4%	0.069
M1	Any exposure (≥1)	Physicians	+0.046	[−1.788, 1.880]	—	0.966
M1	Any exposure (≥1)	Turnover	−0.087	[−0.364, 0.190]	—	0.266
M2	Low-dose (1–4)	None	−0.335*	[−0.618, −0.053]	−9.5%	—
M2	Low-dose (1–4)	Physicians	not estimable (singular matrix)			
M2	Low-dose (1–4)	Turnover	not estimable (singular matrix)			
M2	High-dose (first interaction)	None	−0.365^a^	[−0.609, −0.120]	−9.3%	0.006
M2	High-dose (first interaction)	Physicians	−0.850	[−1.769, 0.069]	—	0.589
M2	High-dose (first interaction)	Turnover	−0.154	[−0.462, 0.154]	—	0.302
M2b	High-dose (threshold onset)	None	−0.266*	[−0.486, −0.047]	−6.8%	0.001
M2b	High-dose (threshold onset)	Physicians	−0.554	[−1.351, 0.242]	—	0.122
M2b	High-dose (threshold onset)	Turnover	+0.040	[−0.275, 0.355]	—	0.074
M3	High vs. low-dose	None	+0.142	[−0.105, 0.389]	—^a^	0.003^b^
M3	High vs. low-dose	Physicians	+0.130	[−0.200, 0.461]	—^a^	0.145^b^
M3	High vs. low-dose	Turnover	+0.149	[−0.199, 0.496]	—^a^	0.035^b^

*p < 0.05. Pre-trends *p* is the joint *χ*2 test on ten pre-treatment event-study coefficients.

a Relative change is not reported for M3, since it compares two already-exposed groups directly rather than measuring change from a pre-treatment baseline; for M1/M2/M2b, relative change is computed against each row's own group-specific pre-treatment mean referral rate (Table 1).

b For M3, whose comparison group is the low-dose group rather than never-treated units, pre-trends *p* tests whether high- and low-dose units were on parallel trends with each other prior to the high-dose group crossing the threshold — a different comparison than for M1/M2/M2b, not a test of the same underlying assumption.

The similarity in magnitude between the low- and high-dose estimates together with the null and positive incremental estimate, indicates no evidence that effects increase with cumulative dose: the estimated reduction is more closely associated with program participation rather than with the intensity of engagement. We return to the candidate mechanisms consistent with this pattern in the Discussion.

**Figure 4:**
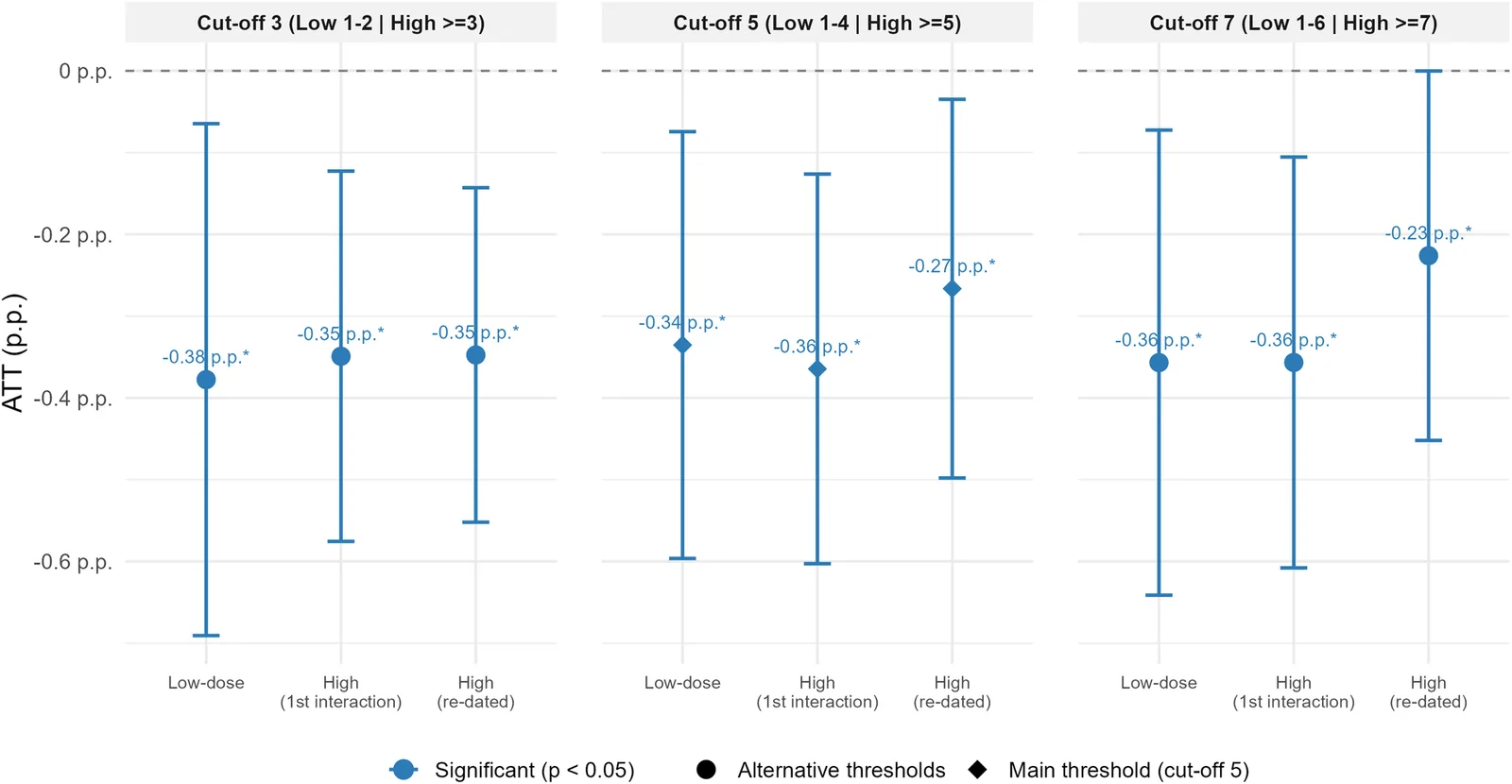
Event-study estimates for the paper's three primary specifications — M1 (any exposure vs. never-treated; green squares), M2 (high-dose vs. never-treated, onset dated at first interaction; orange circles), and M2b (high-dose vs. never-treated, onset re-dated at threshold crossing; blue triangles) — across three covariate specifications: no covariates (top), conditioning on number of physicians (middle), and conditioning on physician turnover rate (bottom). Dotted vertical line marks the boundary between the last pre-treatment period (quarter −1) and treatment onset (quarter 0); horizontal dashed line marks zero. Error bars are 95% confidence intervals based on bootstrap standard errors (999 replications) clustered at the primary care unit level. Each panel uses its own *y*-axis scale, reflecting the substantially wider confidence intervals in the covariate-adjusted specifications (see Results); axis values, not bar heights, should be compared across panels. Estimator: Callaway & Sant'Anna (2021). Sample: Northeast Brazil, excluding Ceará and Bahia; panel observed 2018–Q2 2025, with treatment onset from Q3 2022.

The dynamic evolution of these effects is examined in Figure 4, which overlays event-study estimates for M1, M2, and M2b across all three covariate specifications.

Covariate-adjusted specifications show a different pattern from the no-covariate results above. Conditioning on the number of physicians or on physician turnover substantially widens confidence intervals across all models — standard errors roughly three to four times larger than the corresponding no-covariate estimate (see Figure 4; note that panels use independent *y*-axis scales) — and most estimates lose statistical significance; the low-dose model fails to estimate at all under either covariate specification, due to a singular covariance matrix. This pattern is likely explained by the covariate-adjusted models being estimated on a much smaller, non-randomly-selected subsample: workforce data are available for only 22%–29% of unit-quarters across the three study groups (Table 1), and this coverage is correlated with registry completeness. To confirm this directly, we re-estimated the no-covariate model on the same restricted subsample used by each covariate-adjusted specification: in every case, the estimate shifts substantially and loses significance upon restricting the sample alone, before the covariate enters the model, confirming that subsample selection — not the covariate adjustment itself — drives the attenuation (Supplementary Appendix Table A1).

Formal joint chi-squared tests of pre-treatment event-study coefficients (Figure 4) show that the no-covariate specification fails the parallel-trends test for the dose-differentiated models [M2: *χ*²(10) = 24.84, *p* = 0.006; M2b: *χ*²(10) = 28.51, *p* = 0.001], while the covariate-adjusted specifications, which do satisfy the test (physicians: *p* = 0.589 and *p* = 0.122; turnover: *p* = 0.302 and *p* = 0.074, for M2 and M2b respectively), lack the precision to confirm or rule out an effect. M3 also fails the joint pre-trends test [*χ*²(10) = 26.13, *p* = 0.003], indicating that high- and low-dose units were not on parallel trends with each other before the high-dose group crossed the threshold; we discuss the implications of this for interpreting the incremental estimate in the Discussion. M1 satisfies both criteria simultaneously: its no-covariate estimate is the most precisely estimated in the paper, and its pre-treatment coefficients come closest to satisfying the joint test [*χ*²(10) = 17.23, *p* = 0.069], comfortably satisfied when either covariate is added (*p* = 0.966 and *p* = 0.266).

Accordingly, we treat M1 as our primary specification, and present M2, M2b, and M3 as secondary evidence bearing on mechanism (Supplementary Appendix Table A1). As a further, more formal check on this identification caveat, we applied the “honest” confidence-interval approach of Rambachan and Roth (24) under the relative-magnitude restriction to M1, M2, and M2b (no-covariate specification), targeting the average effect across the reported post-treatment window rather than a single event-time coefficient. All three specifications lose statistical significance once the permitted post-treatment deviation from parallel trends reaches half the magnitude of the largest deviation observed pre-treatment (breakdown M¯ = 0.5 for M1, M2, and M2b alike) (Supplementary Appendix Table A2). By conventional benchmarks (M¯ ≥ 1 is typically considered robust), this indicates fragile rather than robust identification across all three specifications — a caveat we weigh accordingly in the Discussion.

Results were materially unchanged when we excluded units in the worst pre-rollout data-quality tiers (see Study design): estimated effects for M1 and the high-dose specifications remained similar in magnitude and direction across all quality-cutoff scenarios tested, with no evidence that our findings depend on retaining lower-quality units in the sample.

Because the earliest treated cohorts' pre-treatment period spans 2020–2022, coinciding with the COVID-19 pandemic's disruption of Brazilian primary care, we also tested sensitivity to this by restricting the panel to more recent calendar quarters. Results were mixed rather than uniformly favorable to a pandemic-driven explanation. Restricting the panel to Q3 2021 onward worsened the joint pre-trends test for all three specifications [M1: *χ*²(10) = 22.54, *p* = 0.013; M2: *p* = 0.003; M2b: *p* < 0.001]; restricting further to Q1 2022 onward improved it for M1 (*p* = 0.344) and, to a lesser extent, M2 (*p* = 0.046), but left M2b essentially unchanged (*p* = 0.001). The point estimates were stable across both restrictions. This indicates that pandemic-era pre-treatment data contributes to M1's parallel-trends fragility specifically, but is not the primary driver of M2b's — a limitation that persists independent of the pandemic period.

Exposed (low- and high-dose) units had a combined, group-size-weighted mean pre-treatment referral rate of approximately 3.79%, against 5.00% among never-treated units (Table 1). This gap is not small relative to the estimated effect: the M1 no-covariate ATT of −0.356 percentage points corresponds to roughly 30% of the 1.21-percentage-point baseline difference between groups, and implies a relative reduction of approximately 9% in referrals to specialized care. A gap of this magnitude nonetheless means that even a modest departure from parallel trends could account for a non-trivial share of the estimated effect, reinforcing why we weight the formal pre-trends and Honest DiD results, rather than the point estimate alone, in treating M1 as our primary specification.

## 4 Discussion

This study provides quasi-experimental evidence that participation in the TeleNordeste program is associated with a meaningful reduction in referral rates to specialized care. We found no evidence that this association increased with cumulative exposure to tele-interconsultations. This finding is consistent with prior evidence showing that access to specialist input through professional-to-professional synchronous tele-interconsultations is associated with changes in referral patterns, even in the absence of large effect sizes (8, 11).

Two channels could plausibly generate this effect. The first is an immediate reorganization of care flows: a case that would otherwise have been referred is instead resolved through the tele-interconsultation itself. The second is workplace learning: repeated exposure to specialist reasoning gradually builds diagnostic confidence and case-management capacity, an effect that should compound with the number of interactions. Our design was intended, in part, to adjudicate between these channels using the dose-response and incremental (M3) specifications. It does not allow us to do so. The low- and high-dose estimates are of similar magnitude, and the incremental comparison between them is null and wrong-signed. While an immediate reorganization of care flows offers a plausible and sufficient explanation for the effect we estimate, our data do not allow us to confirm whether workplace learning also contributes — a conclusion we hold loosely in either direction, given the parallel-trends caveat attached to the dose-differentiated specifications, which we show is not fully attributable to the COVID-19 pandemic period (see Results).

We tested this gradient using a categorical rather than a fully continuous treatment-intensity estimator (e.g., Callaway, Goodman-Bacon and Sant'Anna's continuous extension of staggered DiD), both for interpretability given the extreme right-skew in cumulative interactions (see Study design) and because continuous-treatment extensions of staggered DiD remain comparatively new, with fewer established diagnostics — including compatibility with the parallel-trends sensitivity analysis below — than the categorical framework used throughout this paper. Within this framework, M3 is our most direct dose-gradient test, though its own comparison groups — high- and low-dose units — do not satisfy parallel trends with each other prior to the high-dose group crossing the threshold (see Results), the same identification caveat attached to M2 and M2b. With that caveat in mind, M3 yields no evidence of a dose gradient, a conclusion further supported by the similar absolute magnitude of the low- and high-dose estimates in Table 2. We therefore report the effect on referral rates as our primary finding and treat the mechanism as an open question, rather than attributing it to either channel.

A magnitude-based argument is sometimes used to favor a learning interpretation over pure substitution, since tele-interconsultations represent a very small share of total consultations (0.445%). We tested this directly: bounding calculations based on program data show that substitution alone plausibly accounts for anywhere from roughly 30% to essentially all of the estimated effect, depending on what share of tele-interconsultations represent genuinely new cases rather than scheduled follow-ups — a distinction our data cannot resolve. We therefore do not treat the effect's magnitude as informative about mechanism in either direction. More broadly, referrals are shaped by factors — service availability, regulatory arrangements, patient expectations, institutional incentives — that telehealth does not directly affect; small but systematic reductions may reflect meaningful refinements in clinical judgment rather than structural shifts in care pathways, consistent with reviews showing that specialist-to-primary-care tele-interconsultations tend to generate incremental rather than dramatic changes in utilization (7).

If a learning channel is contributing to the effect we estimate, the workforce profile characteristic of primary care in Brazil offers a plausible reason why it might be relevant here specifically. PHC units, particularly in underserved regions, are disproportionately staffed by recently graduated, non-specialist physicians: approximately 70% had graduated less than five years prior to hire, and only 5% hold a family medicine qualification (25, 26); turnover studies confirm this workforce skews toward young, inexperienced graduates who remain in these settings for short periods (26, 27). For this population — clinically capable but early in their professional development, in resource-constrained environments — repeated exposure to specialist reasoning could plausibly offer a structured pathway for on-the-job learning; our data, however, cannot confirm that this channel, rather than immediate case resolution, is what drives the estimated effect.

The regional context further shapes interpretation. Persistent inequalities in access to health services, physician distribution, and specialist availability in the Northeast create conditions under which telehealth may play a supportive but necessarily limited role (15, 16, 23); it cannot substitute for structural investments in workforce supply and service capacity, but may help mitigate clinical uncertainty at the margin in underserved settings. The concentration of use in endocrinology, neurology, and cardiology is consistent with international and national evidence on telemedicine's role in chronic disease management and diagnostic decision-making in primary care (2, 13); descriptive studies of TeleNordeste's implementation document similar specialty patterns, with neurological conditions — including pediatric cases — representing a significant share of tele-interconsultations in states where neurological specialist supply is critically low (18, 19).

Two limitations of the data and study design are central to interpreting our results, since they bear directly on our inability to distinguish the two mechanisms above. The first concerns zero-referral quarters. Referral rates derived from SISAB appear low relative to the literature (13, 28), and heterogeneity in registration practices across Brazilian municipalities is well documented, particularly where administrative capacity is limited (22, 23). A quarter with zero recorded referrals is therefore ambiguous — it may reflect a quarter in which every case was genuinely resolved without referral (arguably the strongest possible signature of flow reorganization), or incomplete registration unrelated to clinical practice. We excluded the 431 units for which this pattern was total and persistent (zero referrals in every observed quarter alongside otherwise-normal consultation volumes), but retained isolated zero-referral quarters within otherwise-reporting units, since excluding them would risk discarding exactly the observations most informative about a genuine effect. This choice means our estimates could be biased in either direction by registration variation correlated with treatment status — inflating the effect if concentrated among high-dose units, masking it if concentrated among comparison units — and we cannot determine the direction or magnitude of this potential bias with the present data.

The second concerns the unit of analysis. Our analysis is conducted at the primary care unit level, while workplace learning operates, in the first instance, at the level of the individual physician — the actual target of the intervention. Flow reorganization is less clearly tied to the individual, since triage routines or case-discussion practices could shift at the team level; this channel may therefore operate at the unit level, the individual-physician level, or both. Our administrative data include no physician identifier linking clinicians to specific tele-interconsultations, so we cannot determine whether an estimated unit-level effect reflects broad staff engagement or a small subset of participating clinicians, nor track physicians as they move between units. High turnover and multi-site practice introduce two further sources of attenuation: a unit may remain classified as exposed after the physicians who engaged with the program have left, while a physician's telehealth experience may transfer to a unit classified as untreated. Both would be resolved by physician-level, rather than unit-level, data.

Should the TeleNordeste program continue, we recommend three changes to routine data collection that would directly address these limitations: (i) a physician-level identifier linking clinicians to the tele-interconsultations they participate in and to the consultations and referral decisions they make, moving the analysis to the level at which both candidate mechanisms actually operate; (ii) a lightweight audit or validation process for referral registration, to distinguish genuine zero-referral quarters from underreporting; and (iii) a case-level flag distinguishing new from follow-up tele-interconsultations, to resolve rather than merely bound the magnitude argument above. Any of these changes alone would materially improve future evaluations' ability to adjudicate between the mechanisms we can only describe, but not distinguish, here.

Taken together, these results suggest that specialist-support telehealth should be understood less as a transformative intervention and more as a modest but real contributor to primary care case resolution, operating through a mechanism — or combination of mechanisms — that this study can characterize only partially. Evaluations of telehealth initiatives should not assume that effects, if present, reflect workforce development specifically; programs should be evaluated on the outcomes they demonstrably move, with claims about the underlying behavioral mechanism treated as a separate question requiring its own evidence. Program designs that promote continuity and repeated engagement remain reasonable on other grounds — including the plausibility of a flow-reorganization channel that itself benefits from sustained availability of specialist support — consistent with international guidance on telemedicine implementation (1, 6).

These policy implications must be read alongside caveats about generalizability. The observed effects reflect not only mechanism but also the specific conditions under which TeleNordeste operated: a structured public-private partnership delivered, within the states covered by this analysis, by four hospitals, each with its own specialist teams, clinical protocols, and operational practices; variation in implementation fidelity across institutions may have shaped interaction intensity and quality in ways not captured by administrative data. The analysis also covers seven of the nine Northeast states, limiting geographic representativeness within the region. The findings are most directly applicable to contexts combining a large, territorially dispersed primary care system, limited specialist availability, and a structured telehealth program with sustained engagement — conditions found elsewhere in Brazil and in similarly configured low- and middle-income health systems (1, 29).

By focusing on synchronous tele-interconsultations as embedded clinical interactions, this study contributes to a growing literature examining digital health not only as a tool for expanding access, but as a potential input into how primary care physicians manage and resolve clinical cases. The findings suggest that the value of specialist-support telehealth can extend beyond expanded access, though our design cannot establish how much of the observed effect reflects durable changes in clinical practice as opposed to case-by-case resolution through the tele-interconsultation itself. For health systems seeking scalable strategies to strengthen primary care capacity in underserved regions, this distinction matters for how such programs are designed and evaluated going forward — and resolving it, with the physician-level data this study lacked, is a priority for future work.

## Data Availability

The data analyzed in this study is subject to the following licenses/restrictions: The datasets used in this study are derived from two sources. Primary care consultation and referral data were obtained from the Brazilian Primary Care Health Information System (SISAB), which are publicly available and were accessed via a Freedom of Information request (Lei de Acesso à Informação — LAI). Teleconsultation monitoring data were generated under the TeleNordeste project within the PROADI-SUS program and are held by the participating hospitals, subject to institutional data governance policies. The SISAB microdata are publicly available upon request to the Brazilian Ministry of Health. The TeleNordeste monitoring data supporting the findings of this study are available from the corresponding author upon reasonable request. Requests to access these datasets should be directed to Frederica Padilha: frepadilha@hotmail.com.
