# Supplementary material for: Reducing referrals in primary care through specialist-supported telehealth: quasi-experimental evidence from Northeast Brazil
Source: Front Digit Health. 2026 Sep 9;8:1890968. doi: 10.3389/fdgth.2026.1890968 (PMC13597718; doi:10.3389/fdgth.2026.1890968)
Supplement: Supplementary file 1 [file Supplementaryfile1.docx]

**Appendix**

**Figure A1**

**Appendix Table A1. Decomposing covariate-adjusted attenuation into a subsample-selection component and a covariate-adjustment component**

| **Model** | **Covariate** | **Units / unit-quarters** | **(a) No-covariate, full sample** | **(b) No-covariate, restricted sample** | **(c) Covariate-adjusted, restricted sample** |
| --- | --- | --- | --- | --- | --- |
| M1 | Physicians | 6,209 / 50,998 | −0.356* | −0.083 | +0.046 |
| M1 | Turnover | 6,195 / 50,515 | −0.356* | −0.092 | −0.087 |
| M2 (low-dose) | Physicians | 4,424 / 35,450 | −0.335* | +0.073 | not estimable (singular matrix) |
| M2 (low-dose) | Turnover | 4,413 / 35,093 | −0.335* | +0.045 | not estimable (singular matrix) |
| M2 (high-dose) | Physicians | 5,403 / 44,590 | −0.365* | −0.143 | −0.850 |
| M2 (high-dose) | Turnover | 5,389 / 44,169 | −0.365* | −0.143 | −0.154 |
| M2b (high-dose) | Physicians | 5,403 / 44,590 | −0.266* | +0.021 | −0.554 |
| M2b (high-dose) | Turnover | 5,389 / 44,169 | −0.266* | +0.036 | +0.040 |

*All estimates are ATT (percentage points) from the no-covariate specification unless noted. Column (a) is the no-covariate estimate on the full analytical sample (as reported in Table 2). Column (b) is the no-covariate estimate re-estimated on the same restricted subsample used by the corresponding covariate-adjusted model in column (c) — i.e., holding the sample fixed and only removing the covariate from the model — isolating the effect of subsample selection from the effect of covariate adjustment itself. Column (c) is the covariate-adjusted estimate on that restricted subsample (as reported in Table 2). * p < 0.05.*

**Appendix Table A2. Honest DiD sensitivity to parallel-trends violations (relative-magnitude restriction)**

| **Specification** | **M̄** | **CI type** | **95% CI** | **Significant** |
| --- | --- | --- | --- | --- |
| **M1 (any exposure)** | **0.0** | **Original (no relaxation)** | **[−0.632, −0.153]** | **Yes** |
| M1 (any exposure) | 0.0 | Honest (relative magnitude) | [−0.618, −0.173] | Yes |
| M1 (any exposure) | 0.5 | Honest (relative magnitude) | [−1.410, 0.618] | No |
| M1 (any exposure) | 1.0 | Honest (relative magnitude) | [−2.350, 1.560] | No |
| M1 (any exposure) | 1.5 | Honest (relative magnitude) | [−2.450, 2.450] | No |
| M1 (any exposure) | 2.0 | Honest (relative magnitude) | [−2.450, 2.450] | No |
| M2 (onset = 1st interaction) | 0.0 | Original (no relaxation) | [−0.685, −0.137] | Yes |
| M2 (onset = 1st interaction) | 0.0 | Honest (relative magnitude) | [−0.650, −0.198] | Yes |
| M2 (onset = 1st interaction) | 0.5 | Honest (relative magnitude) | [−1.670, 0.820] | No |
| M2 (onset = 1st interaction) | 1.0 | Honest (relative magnitude) | [−2.800, 2.010] | No |
| M2 (onset = 1st interaction) | 1.5 | Honest (relative magnitude) | [−2.800, 2.800] | No |
| M2 (onset = 1st interaction) | 2.0 | Honest (relative magnitude) | [−2.800, 2.800] | No |
| M2b (onset re-dated) | 0.0 | Original (no relaxation) | [−0.624, −0.034] | Yes |
| M2b (onset re-dated) | 0.0 | Honest (relative magnitude) | [−0.577, −0.091] | Yes |
| M2b (onset re-dated) | 0.5 | Honest (relative magnitude) | [−1.610, 0.942] | No |
| M2b (onset re-dated) | 1.0 | Honest (relative magnitude) | [−2.890, 2.220] | No |
| M2b (onset re-dated) | 1.5 | Honest (relative magnitude) | [−3.010, 3.010] | No |
| M2b (onset re-dated) | 2.0 | Honest (relative magnitude) | [−3.010, 3.010] | No |

*M̄ (Mbar) bounds the post-treatment violation of parallel trends relative to the largest pre-treatment violation observed; M̄ = 0 corresponds to no relaxation. "Original" is the conventional (non-robust) confidence interval at M̄ = 0. l_vec is set to an equal-weighted average across all reported post-treatment event-times (quarters 0 through 9), approximating the overall post-treatment effect reported in Table 2 rather than a single event-time. Point estimates and confidence intervals in this table differ modestly from Table 2's, reflecting both this averaging and the analytic (non-bootstrap) standard errors required by the honest_did() procedure.*
